# Retinoic Acid Activates Two Pathways Required for Meiosis in Mice

**DOI:** 10.1371/journal.pgen.1004541

**Published:** 2014-08-07

**Authors:** Jana Koubova, Yueh-Chiang Hu, Tanmoy Bhattacharyya, Y. Q. Shirleen Soh, Mark E. Gill, Mary L. Goodheart, Cathryn A. Hogarth, Michael D. Griswold, David C. Page

**Affiliations:** 1 Whitehead Institute, Cambridge, Massachusetts, United States of America; 2 Department of Biology, Massachusetts Institute of Technology, Cambridge, Massachusetts, United States of America; 3 Howard Hughes Medical Institute, Whitehead Institute, Cambridge, Massachusetts, United States of America; 4 Center for Reproductive Biology, School of Molecular Biosciences, Washington State University, Pullman, Washington, United States of America; Stowers Institute for Medical Research, United States of America

## Abstract

In all sexually reproducing organisms, cells of the germ line must transition from mitosis to meiosis. In mice, retinoic acid (RA), the extrinsic signal for meiotic initiation, activates transcription of *Stra8*, which is required for meiotic DNA replication and the subsequent processes of meiotic prophase. Here we report that RA also activates transcription of *Rec8*, which encodes a component of the cohesin complex that accumulates during meiotic S phase, and which is essential for chromosome synapsis and segregation. This RA induction of *Rec8* occurs in parallel with the induction of *Stra8*, and independently of *Stra8* function, and it is conserved between the sexes. Further, RA induction of *Rec8*, like that of *Stra8*, requires the germ-cell-intrinsic competence factor *Dazl*. Our findings strengthen the importance of RA and *Dazl* in the meiotic transition, provide important details about the *Stra8* pathway, and open avenues to investigate early meiosis through analysis of *Rec8* induction and function.

## Author Summary

The transition from mitosis to meiosis is a defining feature of germ cells, the precursors of eggs and sperm. In mice, retinoic acid (RA), a vitamin A derivative, induces expression of the gene *Stra8*, which in turn is required for the first critical steps of meiosis. The timing of *Stra8* expression in mammalian germ cells is influenced by an RA-degrading enzyme, CYP26B1, that is normally expressed in fetal testes to delay meiosis in males. It is unknown if *Stra8* is RA's only meiosis-inducing target in germ cells or if other such genes are regulated by RA independently of *Stra8*. To investigate this question, we generated two lines of mice: *Cyp26b1* mutants and *Stra8* mutants. Our genetic experiments comparing germ cell development in these two mutants revealed a new RA target, *Rec8*. We demonstrate that *Rec8* upregulation by RA occurs in the same temporal and spatial manner as *Stra8*, but *Rec8* expression is independent of *Stra8*. *Rec8*, like *Stra8*, plays a critical role during early meiotic processes, suggesting that RA induces meiosis in at least two independent pathways. These findings expand our understanding of the gene regulatory network involved in meiotic initiation in mammals.

## Introduction

Most eukaryotes reproduce sexually, with life cycles that display an alternation of diploid and haploid phases. The generation of haploid cells from diploid cells is achieved through meiosis, featuring a single round of DNA replication (meiotic S) followed by two rounds of division (meiosis I and meiosis II).

In all sexually reproducing organisms, including fungi, plants, and animals, cells of the germ line activate the meiotic program when conditions are opportune and appropriate to the species' reproductive strategies. In yeast, for example, the meiotic program is initiated only when diploid cells are starved for nutrients and cannot proliferate. In mammals, the meiotic program is initiated only after the specialized cells of the germ line have migrated to the gonad. The timing of mammalian meiotic initiation differs dramatically between the sexes. In males, meiotic initiation does not commence until a spermatogonial stem cell population has been established, well after birth. In females, meiosis is initiated shortly after the germ cells have entered the gonad, during fetal development.

In mice, the published data are consistent with a model whereby an extrinsic meiosis-initiating signal – retinoic acid (RA) – induces transcription and expression of a single meiotic factor – *Stra8* – which in turn governs the meiotic program [1]–[4]. In the ovary, induction of *Stra8* in fetal germ cells expressing *Dazl*, an intrinsic factor, is required for meiotic DNA replication and the subsequent events of meiotic prophase [2], [5], [6]. In fetal testes, this process is temporarily blocked: CYP26B1, a cytochrome p450 enzyme, degrades RA, preventing expression of *Stra8* and thus precluding meiotic initiation [1], [3], [7]. After birth, RA induces *Stra8* in testicular germ cells, leading to meiotic initiation [3], [4].

Although the currently accepted model in mice postulates that RA induction of *Stra8* may be necessary and sufficient for meiotic initiation [8], evidence suggests that other, independent factors are also at play: germ cells in *Stra8*-deficient fetal ovaries express *Rec8* [2], encoding a meiosis-specific component of the cohesin complex. *Rec8* is required for completion of sister chromatid cohesion, proper synapsis, and chiasmata formation [9], [10]. We decided to examine how *Rec8* expression is regulated during the meiotic transition and whether RA plays a role in its expression. Our investigation proceeded by first comparing the patterns and regulation of *Rec8* and *Stra8* expression and then exploring important differences with respect to their roles in driving meiotic initiation. We discovered that RA activates meiosis in two independent ways, both of which require *Dazl* expression in the germ cells.

## Results

### *Rec8*, like *Stra8*, is expressed in an anterior-to-posterior wave in fetal ovaries

**Figure 1:**
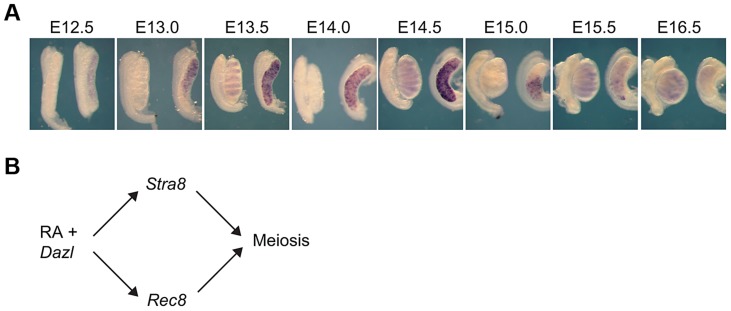
In fetal ovaries, *Rec8* is expressed in an anterior-to-posterior wave. A) *Rec8* expression pattern from E12.5–E16.0 in fetal gonads. B) Proposed model: RA signaling regulates meiotic initiation in mouse germ cells in parallel pathways through *Stra8* and *Rec8*. In all panels, testes are at left and ovaries at right.

We first sought to investigate how *Rec8* expression is initiated in the germ cells of fetal ovaries. If *Rec8* is regulated like *Stra8* and other early meiotic markers, it should initiate expression in an anterior-to-posterior pattern between E12.5 and E16.5 [5], [11], [12]. Using whole mount *in situ* hybridization, we discovered that *Rec8* expression does unfold this way from E13.0 to E16.0 (Figure 1A). These findings suggested that *Rec8*, like *Stra8*, could be a target of RA signaling. Furthermore, since *Dazl* expression is required for ovarian germ cells to respond to RA signaling, perhaps, as with *Stra8* expression, expression of *Rec8* requires both DAZL and RA. We tested this new model (Figure 1B) in fetal ovaries, fetal testes and adult testes.

### RA induces *Rec8* in fetal ovaries

**Figure 2:**
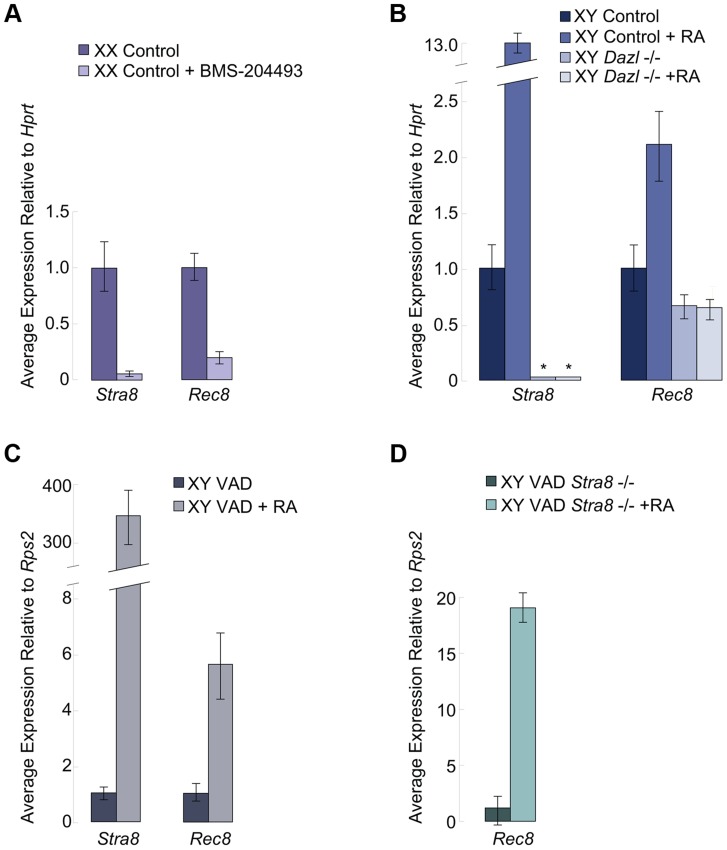
In fetal ovaries and postnatal testes, *Rec8* is a target of RA signaling. Quantitative RT-PCR analyses of A) *Stra8* and *Rec8* transcription in E12.5 ovaries cultured in control medium or with pan-RAR inhibitor added, B) *Stra8* and *Rec8* transcription in E14.5 control and *Dazl-*deficient testes cultured in control medium or with RA added (*Stra8* is undetectable in *Dazl*−/−; indicated by asterisks), C) *Stra8* and *Rec8* expression in RA-restored or control adult VAD testes compared to pre-injection, contralateral testes, and D) *Rec8* expression in *Stra8*-deficient VAD adult testes, without and with RA restoration.

We examined if RA signaling was required for *Rec8* expression in the germ cells of fetal ovaries. We harvested ovaries at E12.5 and cultured them for two days in the presence of the RA receptor pan-antagonist BMS-204493 and then evaluated expression of both *Stra8* and *Rec8* using quantitative RT-PCR. BMS-204493 antagonizes all three RAR isotypes [13] and prevents RA signaling in fetal ovaries without killing the germ cells. We discovered that BMS-204493 dramatically lowered *Rec8* expression, similar to *Stra8* (Figure 2A), indicating that, in wild-type fetal ovaries, RA signaling is required for the germ cells to express *Rec8*. Taking these results together with our laboratory's previous finding that *Stra8*-deficient fetal ovaries express *Rec8* [2], we conclude that RA induces *Rec8* in fetal ovaries independently of *Stra8*.

### In fetal testes, RA-mediated upregulation of *Rec8* requires *Dazl*

We next considered whether RA regulation of *Rec8* expression resembles that of *Stra8* in other respects. Germ cells in wild-type fetal testes express *Stra8* when exposed to high levels of exogenous RA [3], but germ cells in *Dazl*-deficient testes do not [6]. Thus, during meiotic initiation, the germ cells must express *Dazl* in order to respond to RA signaling. We tested whether RA-mediated upregulation of *Rec8* expression similarly requires *Dazl*. We used quantitative RT-PCR to compare *Rec8* expression levels in E12.5 *Dazl-*deficient testes cultured for two days with or without RA added to the medium (Figure 2B). We found that, unlike *Stra8*, *Rec8* is expressed, albeit at very low levels, in wild-type and *Dazl*-deficient testes. However, similarly to *Stra8*, *Rec8* expression was significantly upregulated by RA treatment in wild-type but not *Dazl*-deficient testes (Figure 2B). Thus RA-induced upregulation of *Rec8* in embryonic testes depends on *Dazl*.

### RA induces *Rec8* expression in adult testes independently of *Stra8*

RA also regulates *Stra8* expression and meiotic initiation in germ cells of postnatal testes [3], [4]. We examined whether *Rec8* followed a similar pattern to *Stra8* here as well. Since retinoic acid is a metabolite of vitamin A, vitamin A-deficient (VAD) mice can be used to evaluate the effects of dramatically reduced RA signaling on postnatal testes. We removed testes from several vitamin A-deficient adult males and VAD males with restored RA signaling (24 hours post RA injection) and evaluated *Rec8* and *Stra8* transcripts by quantitative RT-PCR. Like *Stra8*, *Rec8* transcription was dramatically increased 24 hours after injection of RA (Figure 2C). These results demonstrate that RA regulates *Rec8* transcription in adult testes *in vivo*, as in fetal ovaries; this signaling event is shared between the sexes.

To test whether this *Rec8* upregulation in postnatal testes was *Stra8-*dependent, we examined *Rec8* expression in *Stra8-*deficient, VAD testes before and after injection of RA. While the *Stra8-*deficient, RA-deficient VAD testes expressed very little *Rec8*, restoration of RA resulted in dramatically increased expression of *Rec8* (Figure 2D). Thus, as in fetal ovaries, RA induces *Rec8* expression in postnatal testes independently of *Stra8*.

### RA induces *Rec8* expression in *Cyp26b1-*deficient fetal testes independently of *Stra8*

**Figure 3:**
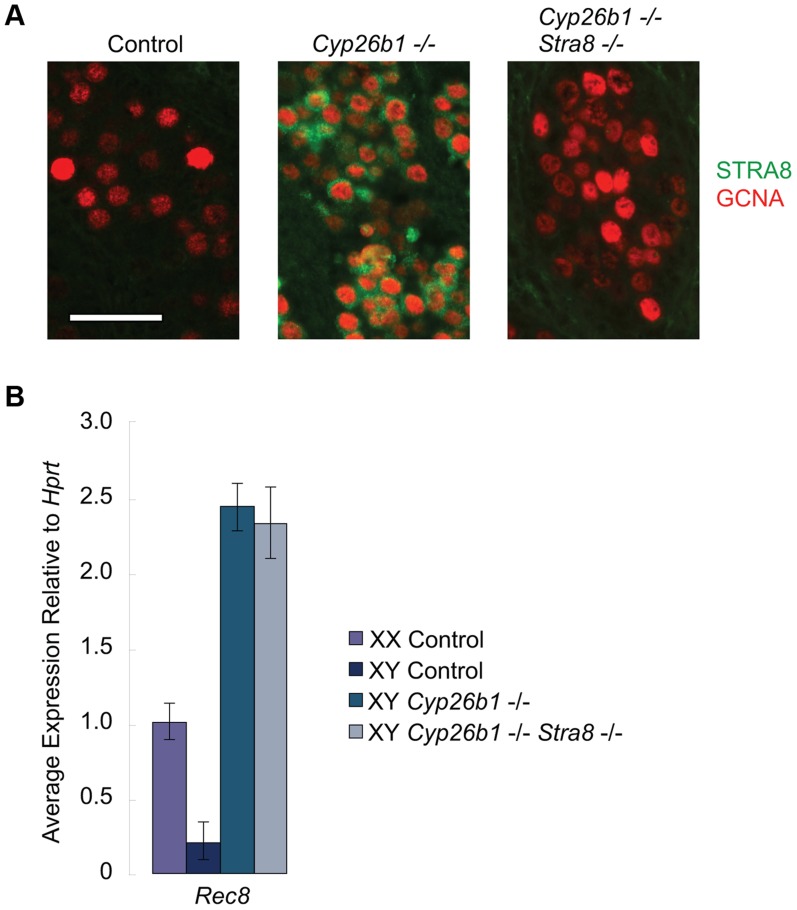
In *Cyp26b1-*deficient/*Stra8-*deficient fetal testes, *Rec8* is induced by RA signaling. A) Fluorescent immunohistochemical staining for STRA8 protein (green) and GCNA (red) in E15.5 testes of the indicated genotypes (400×). Scale bar: 50 µm. B) Quantitative RT-PCR analysis of *Rec8* transcription in E14.5 gonads of the indicated genotypes.

Germ cells in *Cyp26b1-*deficient fetal testes express *Stra8* and several other early meiotic factors at the same time as they do in fetal ovaries because of uninhibited RA signaling [1], [7], [14](Figure S1). However, whether STRA8 protein is expressed and, if so, whether it influences other early meiotic factors has not been determined. We developed a system of single- and double-mutant mice with which to analyze *in vivo* the effects of RA signaling on germ cells in the presence and absence of STRA8. We found that STRA8 protein is expressed in *Cyp26b1*-deficient fetal testes but not in double-mutant *Cyp26b1*-deficient/*Stra8-*deficient testes (Figure 3A). We then assayed *Rec8* expression in single- and double-mutant fetal testes using quantitative RT-PCR. In both cases, *Rec8* expression is higher than in wild type, achieving similar levels in single- and double-mutant samples (Figure 3B). High expression levels in the double mutant indicate that RA induction of *Rec8* in *Cyp26b1-*deficient fetal testes is independent of *Stra8*.

### DNA replication, DNA double-strand break formation, and upregulation of *Dmc1* are all dependent on STRA8 induction in *Cyp26b1-*deficient fetal testes

In our studies above, we have established that RA regulates *Rec8*, and that it does so in parallel to its other known target, *Stra8*, in fetal ovaries, adult testes and in *Cyp26b1-*deficient fetal testes (Figure 1B). Drawing on the comparative model we used to examine *Rec8* expression in fetal testes, we explored whether RA regulates other early meiotic factors in parallel to *Stra8*.

**Figure 4:**
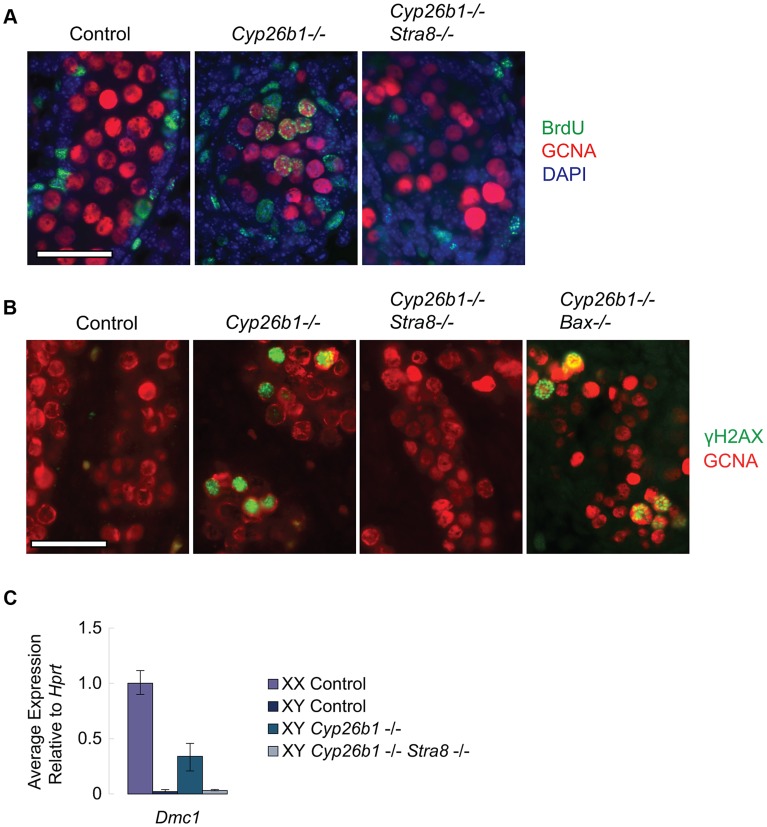
In *Cyp26b1-*deficient testes, STRA8 induces *Dmc1* expression, DNA replication and DNA double-strand break formation. A) Fluorescent immunohistochemical staining for BrdU (green) and GCNA (red) in E16.5 testes of the indicated genotypes (400×). Scale bar: 50 µm. B) Fluorescent immunohistochemical staining for γH2AX protein (green) in E15.5 testes of the indicated genotypes (400×). Scale bar: 50 µm. C) Quantitative RT-PCR analysis of *Dmc1* transcription in E14.5 gonads of the indicated genotypes.

We first tested whether ectopic RA signaling is sufficient to drive DNA replication in germ cells of fetal testes, and, if so, whether this effect is also mediated through STRA8. The thymidine analog 5-bromo-2-deoxyuridine (BrdU) can be incorporated into newly synthesized DNA during S phase. We injected BrdU into pregnant females, dissected E16.5 fetal gonads and immunostained them with anti-GCNA (a germ cell marker) and anti-BrdU antibodies. In wild-type animals, testicular germ cells have arrested in G0/G1 by E16.5. We can therefore detect ectopic germ cell proliferation in response to STRA8 upregulation by assaying for ongoing DNA replication in E16.5 fetal gonads. BrdU incorporation was evident in germ cells of *Cyp26b1*-deficient testes (Figure 4A), consistent with transition towards meiosis. In contrast, GCNA-positive germ cells of double-mutant *Cyp26b1*-deficient/*Stra8-*deficient testes were uniformly negative for BrdU at E16.5. We conclude that the DNA replication observed in germ cells of *Cyp26b1-*deficient fetal testes at E16.5 depends on and is mediated through STRA8 (Figure 4A).

We then determined if RA is sufficient in fetal testes to induce DNA double strand breaks (DSBs), which are required for meiotic recombination [15]–[19], and if the generation of these DSBs is mediated through STRA8 induction. We assayed for the presence of γH2AX, a phosphorylated histone variant that localizes to DSBs, by immunostaining at E15.5, when DSBs are first observed [20]. *Cyp26b1*-deficient testes displayed many germ cells positive for γH2AX, suggesting that DSBs are induced by RA (Figure 4B). In contrast, we rarely observed γH2AX-positive germ cells in double-mutant *Cyp26b1*-deficient/*Stra8-*deficient testes (Figure 4B). This result suggests that the induction of DSBs in *Cyp26b1-*deficient testes is driven by ectopic RA and STRA8.

Since DSBs arise not only during meiotic recombination but also during apoptosis [21], and apoptosis has been reported in *Cyp26b1-*deficient testes [7], we tested whether γH2AX-positive germ cells observed in *Cyp26b1-*deficient testes represent meiotic and not simply apoptotic events. We first generated double mutant *Cyp26b1*-deficient/*Bax-*deficient embryos. *Bax* is a proapoptotic gene, and its deletion has been shown to suppress apoptosis in germ cells [14], [22], [23](Figure S2). Staining in double-mutant *Cyp26b1*-deficient/*Bax*-deficient testes revealed many γH2AX-positive germ cells (Figure 4B), confirming that most γH2AX-positive germ cells observed in *Cyp26b1*-deficient testes represent meiotic rather than apoptotic DNA DSBs. Formation of meiotic DNA DSBs thus represents another portion of the meiotic pathway that is STRA8-mediated.

Meiotic DSBs are processed by DMC1, an ortholog of the bacterial strand exchange protein RecA, which commences expression early during meiotic initiation. We compared the effects of RA on *Dmc1* expression in *Cyp26b1-*deficient testes and in double-mutant (*Cyp26b1-*deficient/*Stra8-*deficient) testes. The *Cyp26b1*-deficient testes displayed increased levels of *Dmc1*, while levels of *Dmc1* in double-mutant testes were similar to controls (Figure 4C). Thus, RA is sufficient to drive *Dmc1* expression in fetal testes *in vivo*, but this induction requires mediation by STRA8.

In summary, it appears that RA induction of STRA8 in fetal testes is required for all of the above-tested markers/processes during early meiosis, with the notable exception of RA-regulated *Rec8* expression.

### *Stra8* and *Dmc1* are expressed independently of *Rec8*

**Figure 5:**
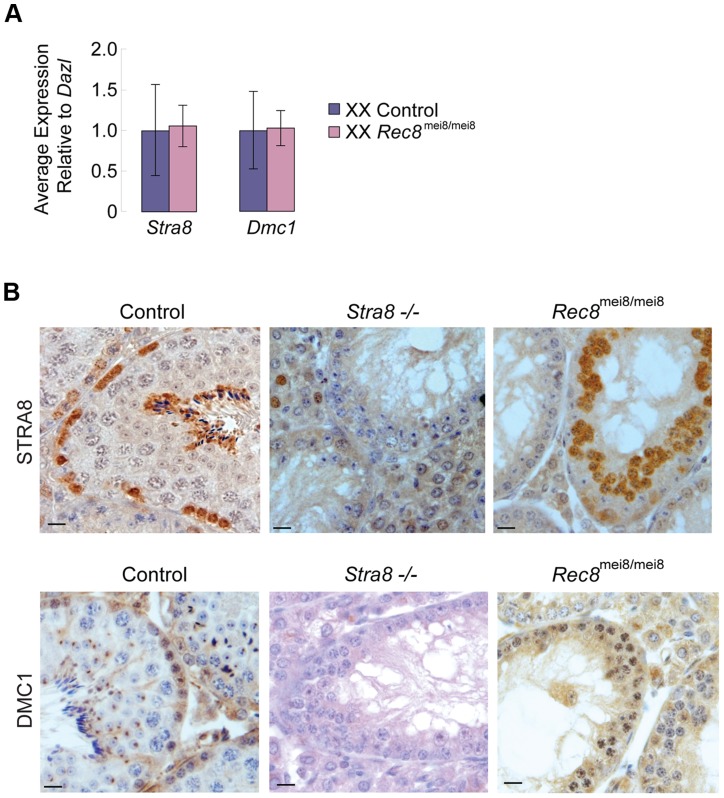
*Stra8* and *Dmc1* expression in male and female germ cells is independent of *Rec8*. A) Quantitative RT-PCR analysis of *Stra8* and *Dmc1* transcription in E13.5 *Rec8*-deficient and control ovaries. B) Colorimetric immunohistochemical staining for STRA8 and DMC1 proteins in control, *Stra8−/−*, and *Rec8^mei8/mei8^* adult testes. Scale bar: 10 µm.

To exclude the possibility that induction of *Stra8* and its downstream target *Dmc1* depends on *Rec8* function, we examined *Stra8* and *Dmc1* expression in *Rec8*-deficient (*Rec8^mei8/mei8^*) ovaries and testes [9]. As expected, we found no significant difference in *Stra8* and *Dmc1* expression levels between control and *Rec8-*deficient E13.5 ovaries (Figure 5A). Similarly, we detected STRA8 and DMC1 proteins in both control and *Rec8-*deficient adult testes (Figure 5B). We conclude that RA induction of *Stra8*, and its downstream targets, is independent of and occurs in parallel with RA induction of *Rec8*.

## Discussion

Our findings lead us to conclude that RA plays a broad and encompassing role in regulating and coordinating the transition from mitosis to meiosis in mouse germ cells, in both fetal ovaries and postnatal testes. Surprisingly, RA accomplishes this by independently inducing both *Stra8* and *Rec8*, which both play critical roles in the earliest stages of meiosis. The discovery that RA induction of *Stra8* in *Cyp26b1-*deficient fetal testes mediates DNA replication, DSB formation, and the expression of recombinase *Dmc1* provides critical details about the *Stra8* pathway. Moreover, *Stra8* induction was recently shown to be required for SYCP3 expression in *Cyp26b1-*deficient testes [24]. *Rec8* induction is the first component of the molecular program of meiotic initiation shown to be *Stra8-*independent in mice. Now that *Rec8*'s independent induction has been established, its expression pattern and function invite deeper investigation.

How *Rec8* expression is induced by RA remains elusive. *Stra8's* promoter region contains two putative RA Response Elements (RAREs), suggesting that RA could be turning on this gene directly [25]. A chromatin immunoprecipitation-sequencing (ChIP-Seq) study in embryonic stem cells identified RAR binding sites in both *Stra8* and *Rec8* promoter regions, suggesting that *Rec8* may also be regulated by RA directly [26]. Intriguingly, in the same study, *Dmc1*, which is dependent on STRA8, does not show such RAR binding sites, consistent with *Stra8* and *Rec8* being regulated directly, unlike *Stra8*'s downstream targets.

What purpose does RA upregulation of REC8 serve? It may ensure that *Rec8* is expressed during pre-meiotic S phase so that its product can be incorporated into the meiotic cohesin complex that joins sister chromatids. Indeed, germ cells in *Rec8*-deficient mice later show defects that can be traced to its cohesion function – incorrect synapsis topology and failure at chromosome segregation and chiasmata formation [9], [10]. Recent studies also suggest a role for cohesins in direct regulation of gene expression by novel mechanisms involving DNA looping [27], [28]. It is presently unknown if *Rec8* is a direct transcriptional regulator. However, *Rec8* null animals exhibit partial embryonic lethality and fail to thrive [10], phenotypes hard to reconcile with an exclusive role in germ cell meiotic cohesion.

The mechanisms that govern meiotic initiation have been explored most thoroughly in yeast, and these studies offer interesting parallels to our findings in mice. In both yeast and mice, the decision to initiate the meiotic program is taken prior to pre-meiotic DNA replication [2], [29]. Our finding that RA regulates *Rec8* is consistent with an early role of RA in this transition, since at least in budding yeast, REC8 associates with chromosomes from late G1 phase [30]. In addition, in both yeast and mice, the decision to initiate meiosis requires an extrinsic signal and an intrinsic competence factor [1], [3], [6], [31], [32]. In yeast, the extrinsic signal – nutrient depletion – activates multiple molecular pathways in parallel, and these converge on IME1, which is required for upregulating the expression of meiosis-specific transcripts. However, IME1 is not sufficient to induce meiosis in yeast [33], [34]. Our studies show that, analogously, RA activates at least two pathways by regulating *Stra8* and *Rec8* independently. While many early meiotic processes described so far hinge on STRA8, STRA8 may not be sufficient for meiosis in mice. The search for additional RA targets will likely yield further insights into the networks governing transition from mitosis to meiosis in mammals.

## Materials and Methods

### Ethics statement

All experiments involving mice were approved by the Committee on Animal Care at the Massachusetts Institute of Technology.

### Targeted disruption of the *Cyp26b1* gene

*Cyp26b1-*deficient mice were generated by deleting a 2.9-kb portion of the gene (including exons 4, 5, 6, and the coding region of exon 7) by homologous recombination in embryonic stem (ES) cells (Figure S1). A *Cyp26b1/PGK-Neo* targeting construct was assembled using PCR products amplified with Advantage HF2 polymerase (Clontech) using mouse (C57BL/6J) genomic BAC RP24-470O13 (GenBank Accession AC159337) as template. The targeting construct was linearized and electroporated into v6.5 ES cells [35]. Cells harboring the construct were selected using neomycin (Invitrogen). ES cell colonies were screened by PCR for homologous integration at both the 5′ and 3′ arms of the construct. Clones that tested positive by both PCR assays were confirmed by Southern blot analysis using EcoRV and Nde1 restriction endonucleases.

Correctly targeted ES cell clones were injected into Balb/c or C57Bl/6N blastocysts and transferred to pseudopregnant Swiss Webster females. Germline transmission was obtained with one clone, and the resulting homozygous embryos displayed anomalies of limb, eye, and facial development and died at birth, as previously described [7], [36]. Embryos were genotyped by PCR, (primer sequences available in Note S1).

### Additional mutant mouse strains

Mice carrying the *Dazl^TM1Hgu^* allele [37] were generously provided by Howard Cooke, MRC Human Genetics Unit, Western General Hospital, Edinburgh, UK, and *Dazl-*deficient mice were generated as described previously [6], [38]. *Stra8*-deficient mice were generated as described previously [2], [4]. *Bax-*deficient mice were generated by mating *Bax^tm1Sjk/+^* mice obtained from The Jackson Laboratory (Bar Harbor, ME). *Rec8-*deficient mice were generated by mating *Rec8^mei8/+^* mice [9], which were generously provided by John Schimenti, Cornell University, Ithaca, New York.

### Mouse embryo collection and *in situ* hybridization

Mouse embryos used in whole mount in situ hybridizations and gonad cultures were obtained from matings between CD1 random bred mice (Charles River Labs). Noon of the day when vaginal plug was recorded was considered E0.5. Whole mount in situ hybridizations with the *Stra8* probe were performed as previously described [3], [39]. Digoxigenin riboprobe for *Rec8* was generated by amplifying cDNA fragments by RT-PCR from *Rec8* (NM_020002.2: bases 274–865), and inserting them into TA cloning vector pCR4-TOPO (Invitrogen). Plasmid was linearized with Spe1 or Not1 and transcribed with T7 or T3 respectively to make the antisense and sense probes.

### RT-PCR

For experiments involving *Rec8-*deficient mice, total RNAs were prepared from gonads using the RNeasy plus Micro RNA isolation kit (QIAGEN), and reverse transcription was carried out using the high-capacity cDNA reverse transcription kit (Applied Biosystems). For all other experiments, total RNAs were prepared using TRIzol (Invitrogen) extraction followed by DNase (Ambion) treatment, and reverse transcription was carried out using the RETROscript reverse transcription kit (Life Technologies). The resulting total cDNAs were analyzed quantitatively using SYBR Green PCR reagents (Applied Biosystems) with primers for *Dmc1*, *Rec8*, *Stra8*, or *Dazl*. Expression profiles were tested in triplicate on at least two litters of embryos on an ABI 7500 instrument (Applied Biosystems). Data were analyzed using the comparative Ct (ΔΔCt) method and one-tail, unpaired student T test (significance cutoff p<0.01). Results were normalized to *Rps2* (VAD experiments on adult testis), *Dazl* (*Rec8-*mutant experiments on embryonic ovary), and *Hprt* (all other experiments). Primers were selected from PrimerBank [40] (Note S1).

### Immunofluorescent studies of tissue sections

Fetal gonads were dissected in phosphate buffered saline (PBS), fixed in 4% paraformaldehyde overnight at 4°C, embedded in paraffin and sectioned. Slides were incubated with anti-GCNA IgM (courtesy of G. Enders, undiluted supernatant), anti-STRA8 (Abcam. 1∶100), and anti-phosphoH2A.X (Upstate Cell Signaling Solutions, 1∶250 dilution). Colorimetric staining was performed using ABC reagents (Vector Laboratories) and developed with DAB peroxidase substrate (Vector Laboratories).

Sections were mounted in Vectashield Medium with DAPI (Vector Laboratories), and fluorescent staining was obtained using Texas-Red or FITC-conjugated secondary antibodies (Jackson Immunoresearch Laboratories, 1∶500 dilution).

### Immunohistochemical studies of tissue sections

Adult testes were fixed in Bouin's solution overnight at 4°C, washed with PBS and 70% ethanol, embedded in paraffin, and sectioned at 5 µm thickness. Slides were matured overnight, de-waxed, rehydrated, and heated in 10 mM sodium citrate buffer (pH 6.0) for antigen retrieval. Sections were incubated in 3% hydrogen peroxide for 5 min and blocked in 2.5% normal horse serum (Vector Laboratories) for 80 minutes at room temperature. Later, slides were incubated overnight with anti-STRA8 (Abcam, 1∶500) or anti-DMC1 (Santa Cruz Biotechnology, 1∶50 dilution). The following day, slides were washed three times in PBS and incubated with anti-rabbit ImmPRESS peroxidase reagent (Vector Laboratories) for 30 minutes. The slides were later developed using a DAB substrate kit (Vector Laboratories) for 1 minute. The slides were counterstained with Mayer's hematoxylin for 5 minutes and washed in running water, dehydrated, and mounted with Permount (Fisher Scientific).

### TUNEL analysis

Apoptotic cells were detected in paraffin sections of fetal testes using the Fluorescein *in situ* Cell Death Detection Kit (Roche Applied Science) and mounted in Vectashield Medium with DAPI (Vector Laboratories).

### BrdU incorporation

Pregnant females were injected with 5-bromo-2-deoxyuridine (BrdU) solution (50 mg/kg) at 18.5 days *post coitum*. Six hours later, fetal gonads were dissected. Gonads were then fixed in 4% paraformaldehyde overnight at 4°C, embedded in paraffin, and sectioned. Prior to antibody application, sections were treated with denaturing reagent (3.5N HCl) for 2 min. Incorporated BrdU was detected using anti-BrdU (Accurate Chemical & Scientific Corp., 1∶500 dilution) in anti-GCNA IgM supernatant.

### Mouse fetal gonad culture

Pregnant female mice were sacrificed by cervical dislocation and embryos were removed into PBS solution. After determining tail somite number, fetal ovaries and mesonephroi were dissected. One gonad from each embryo was then placed in a 35 µl droplet of culture media (DME +10% FBS) supplemented with either 5 µM pan-RAR inhibitor BMS-204493 (Bristol-Myers Squibb) or all trans RA (Sigma) dissolved in ethanol in a Petri plate. Control media contained vehicle (ethanol) alone. Petri plates were then inverted and placed within larger plates containing water and incubated at 37°C with 5% CO_2_. Media was replaced after 24 hours. After 48 hours, tissue was removed from media, mesonephroi were dissected off and ovaries were placed individually into TRIzol reagent (Invitrogen). Samples were then processed for quantitative RT-PCR as described above.

### Analysis of *Rec8* expression in vitamin-A-deficient testes

Adult female mice (129/SvJ) were fed a Vitamin-A-Deficient (VAD) diet (Harlan Teklad, Indianapolis) for at least 2 weeks before mating and throughout pregnancy. Their male offspring were fed a VAD diet for 13–14 weeks. In the first experiment with wild-type animals, one testis was removed from each animal and cut into two pieces; one fixed in Bouin's solution for histological assessment of spermatogenesis and the other placed in TRIzol (Invitrogen) for RNA extraction to serve as a pre-injection control in RT-PCR analysis. Incisions were sutured and the animals recovered for 24 h. Three animals with similarly deficient spermatogenesis (as judged by pre-injection testicular histology) were injected with 100 µl of 7.5 mg/ml all-trans retinoic acid (Sigma) in 10% ethanol/90%sesame oil solution. The animals' remaining testes were harvested 24 h after injection. In contrast, both testes were harvested from two *Stra8-*deficient VAD animals at the same time (one was analyzed histologically to confirm depletion) and compared to testes harvested from two RA-restored *Stra8-*deficient animals. Quantitative RT-PCR analysis was performed, in triplicate, using *Stra8* and *Rec8* primers, and *Rps2* was used as a normalization control (primer sequences in Note S1).

## Supporting Information

Figure S1 Targeted disruption of the *Cyp26b1* locus on mouse chromosome 6. A) Homologous recombination removes exons 4, 5, 6 and the coding portion of exon 7, and replaces them with a loxP-PGK-Neo-loxP selection cassette. B) Correctly targeted ES cell clones were confirmed by Southern blot analysis (E, EcoRV; N, Nde1). Positions of 5′ (red) and 3′ (green) probes are shown in part A. C) E14.5 *Cyp26b1*−/− embryos exhibit defects in limb and facial development as previously reported (Yashiro et al., 2004). D) Whole-mount in situ hybridization with *Stra8* probe reveals staining in *Cyp26b1*−/− testes. E) Quantitative real-time PCR shows increase in *Stra8* expression levels in *Cyp26b1*−/− testes.
(PDF)

Figure S2 Immunohistochemical staining for MVH protein (red) and TUNEL staining (green) in E15.5 control ovary and testis, *Cyp26b1*-deficient testis, and double-mutant (*Cyp26b1*-deficient, *Bax*-deficient) testis.
(PDF)

Note S1 Primer sequences for genotyping *Cyp26b1-*deficient mice and RT-PCR analyses.
(DOCX)
