# Supplementary figures and images for: Retinoic Acid Activates Two Pathways Required for Meiosis in Mice
Source: PLoS Genet. 2014 Aug 7;10(8):e1004541. doi: 10.1371/journal.pgen.1004541 (PMC4125102; doi:10.1371/journal.pgen.1004541)

Figure S1

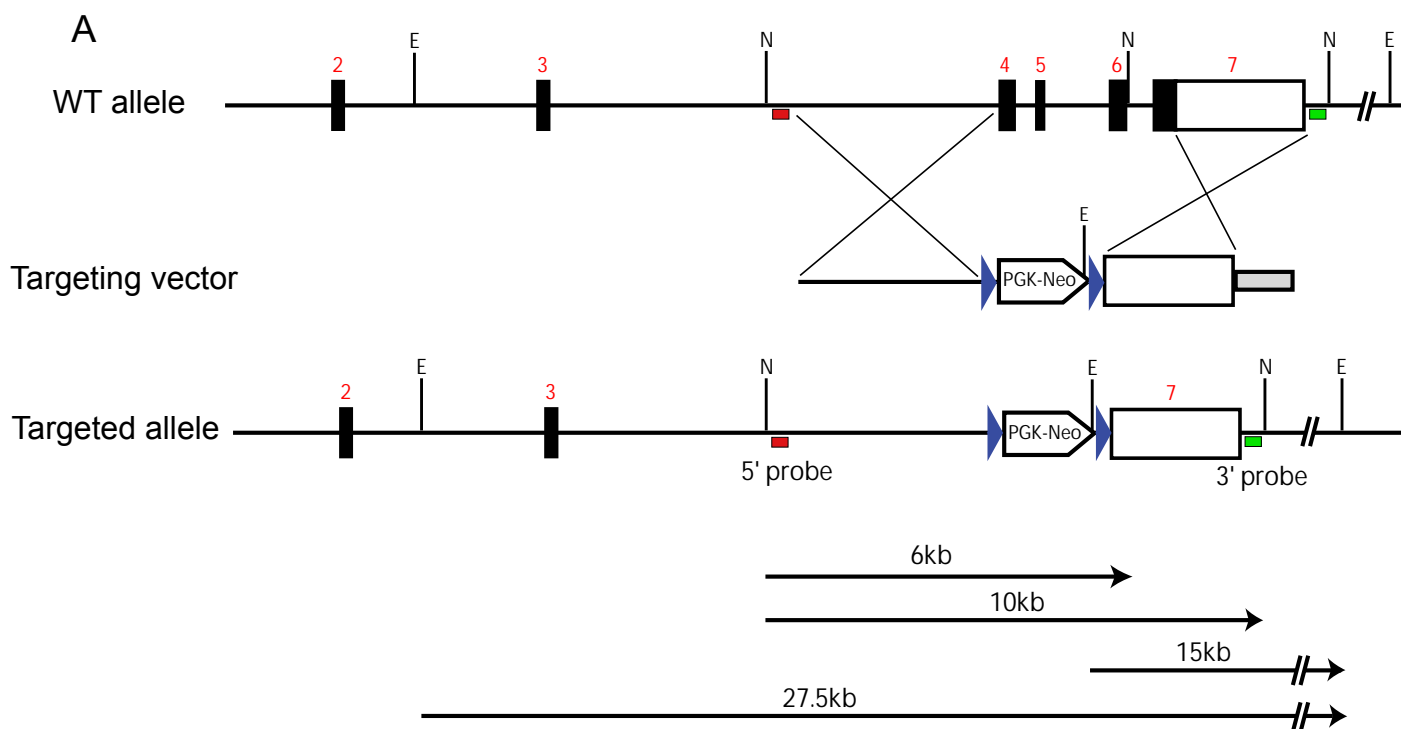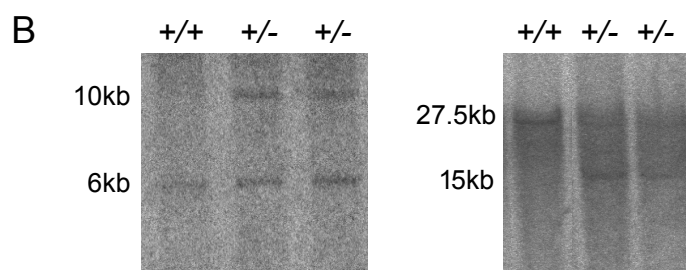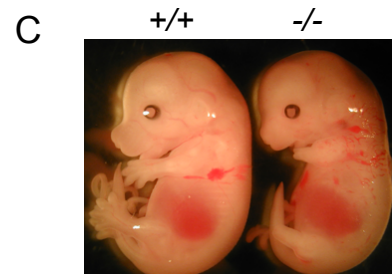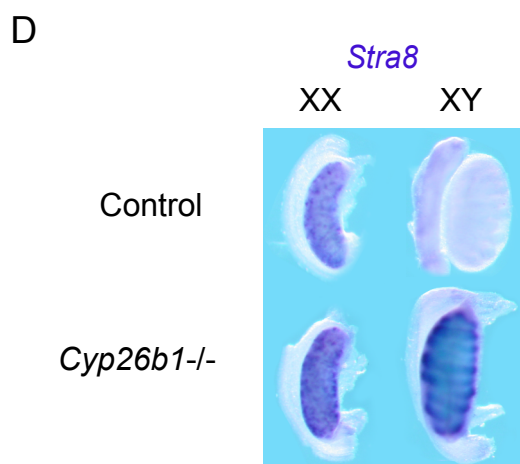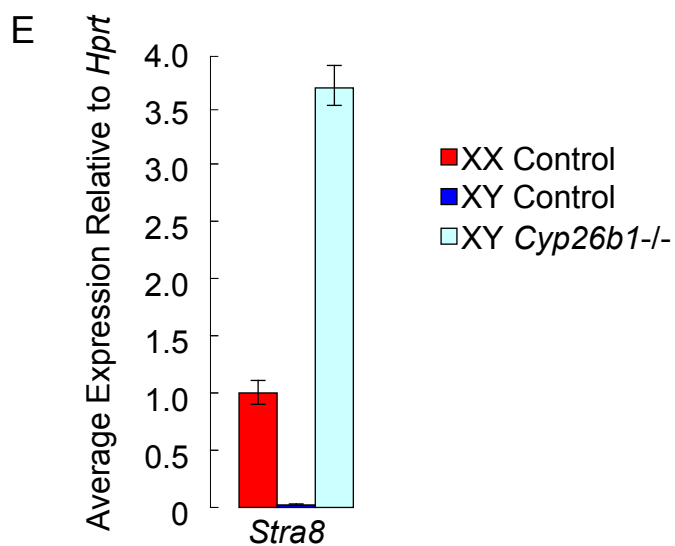

Supplement: Figure S1 — Targeted disruption of the Cyp26b1 locus on mouse chromosome 6. A) Homologous recombination removes exons 4, 5, 6 and the coding portion of exon 7, and replaces them with a loxP-PGK-Neo-loxP selection cassette. B) Correctly targeted ES cell clones were confirmed by Southern blot analysis (E, EcoRV; N, Nde1). Positions of 5′ (red) and 3′ (green) probes are shown in part A. C) E14.5 Cyp26b1−/− embryos exhibit defects in limb and facial development as previously reported (Yashiro et al., 2004). D) Whole-mount in situ hybridization with Stra8 probe reveals staining in Cyp26b1−/− testes. E) Quantitative real-time PCR shows increase in Stra8 expression levels in Cyp26b1−/− testes. (PDF) [file pgen.1004541.s001.pdf]

Figure S2

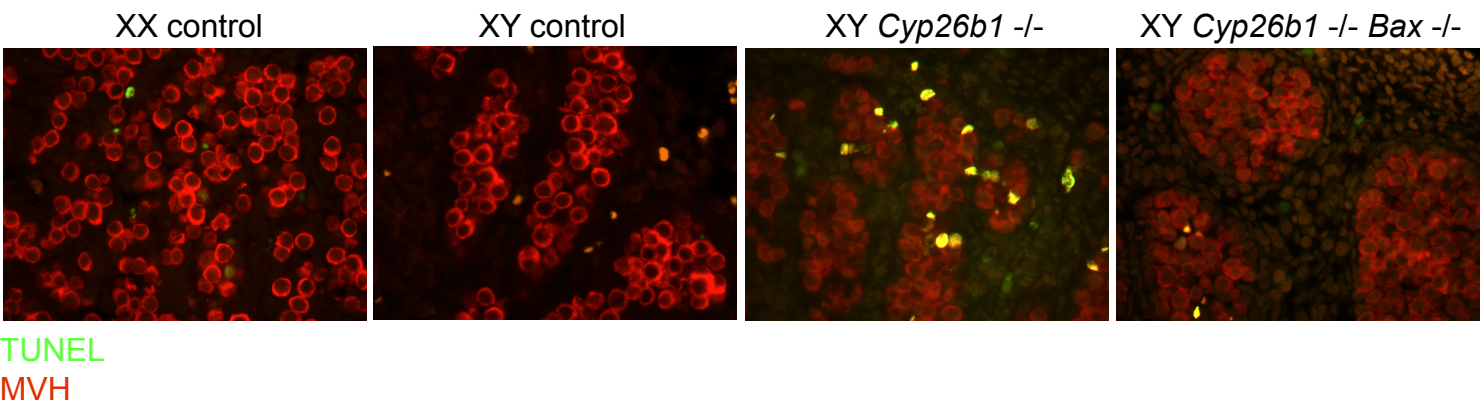

Supplement: Figure S2 — Immunohistochemical staining for MVH protein (red) and TUNEL staining (green) in E15.5 control ovary and testis, Cyp26b1-deficient testis, and double-mutant (Cyp26b1-deficient, Bax-deficient) testis. (PDF) [file pgen.1004541.s002.pdf]
