# Supplementary material for: Retinoic Acid Activates Two Pathways Required for Meiosis in Mice
Source: PLoS Genet. 2014 Aug 7;10(8):e1004541. doi: 10.1371/journal.pgen.1004541 (PMC4125102; doi:10.1371/journal.pgen.1004541)
Supplement: Note S1 — Primer sequences for genotyping Cyp26b1-deficient mice and RT-PCR analyses. (DOCX) [file pgen.1004541.s003.docx]

**Note S1**

Primer sequences:

**Genotyping *Cyp26b1-*deficient mice:**

Embryos were genotyped using the following three

primers: tgccttgggaaaagtactgataacttcg; gactgcaggggaaattctcacatttaca; and gagaagacattctccacaaactgctggt. The wild-type allele gives rise to a PCR product of 1005 bp.

The mutant allele gives rise to a PCR product of 865 bp. In some cases, embryos were

genotyped by Transnetyx (Memphis, TN, USA).

**RT-PCR primers:**

*Hprt-f*: tcagtcaacgggggacataaa; *Hprt-r*: gggggctgtactgcttaaccag (PrimerBank Accession #7305155a1),

*Dmc1-f*: ccctctgtgtgacagctcaac; *Dmc1-r*: ggtcagcaatgtcccgaag

(PrimerBank Accession #6753650a1),

*Rec8-f*: ctacctagcttgcttcttcc; Rec8-r: gcctctaaaaggtgtcgaa

(PrimerBank Accession #31982699a2),

*Stra8-f*: ctgttgccggacctcatgg; *Stra8-r*: tcacttcatgtgcagagatgatg

(PrimerBank Accession #6678173a2);

*Dazl-f*: atgtctgccacaacttctgag; *Dazl-r*: ctgatttcggtttcatccatcct (PrimerBank Accession #31542549a1).

**RT-PCR primers for VAD testes:**

*Stra8* primers amplified a 151-bp product (primers: 5'-

GTTTCCTGCGTGTTCCACAAG-3' and 5'-CACCCGAGGCTCAAGCTTC-3'),

*Rec8* primers

amplified a 106-bp product (primers:5'-AAGAATGCTCAGACAAAGGCCA-3' and 5'-

CGATCTCGCTCAGAGCTTCAGT-3')

and control *Rps2* primers that amplified a 112-bp product

(primers: 5'-CTGACTCCCGACCTCTGGAAA-3' and 5'-GAGCCTGGGTCCTCTGAACA-3').
